# V_15(Gy)_ as a predictor of asymptomatic radiation pneumonitis in patients with lung cancer: A retrospective dosimetric analysis

**DOI:** 10.1002/pro6.70029

**Published:** 2025-09-17

**Authors:** Weixing Ji, Tao Jiang, Zhihan Chen, Gang Chen, Yang Zhang, Shisuo Du

**Affiliations:** ^1^ Department of Radiation Oncology Zhongshan Hospital Fudan University Shanghai China

**Keywords:** Dosimetry, Logistic Models, Lung Neoplasms, Radiation Pneumonitis, Radiotherapy

## Abstract

**Purpose:**

This study aimed to investigate the dose dependency of asymptomatic radiation pneumonitis (aRP) in patients with lung cancer following radiotherapy, focusing on the predictive value of dosimetric parameters.

**Methods:**

This study included 72 patients with primary lung cancer who underwent radiotherapy between January 2019 and June 2022. The patients were divided into an aRP group (*n* = 30) and a non‐RP group (*n* = 42). The physical dose was converted to an equivalent dose using the Linear‐Quadratic (LQ) model, with an α/β value of 3. Three lung structures were defined, and the corresponding dose‐volume histogram parameters were collected. The Mann–Whitney U test was used to compare dose parameters between the two groups, and multivariate logistic regression was performed to remove correlations among different parameters. A logistic function and receiver operating characteristic curve were constructed to predict aRP. This study analyzed the impact of different clinical characteristics on the aRP incidence.

**Results:**

The lungs‐planning target volume (PTV) V_15(Gy)_ was ultimately identified as the best predictive parameter. Significant dose–response relationships were observed, with V_15(Gy)_ achieving an area under the curve of 0.666 ± 0.067 (*P* = 0.017). The optimal cutoff value for lungs‐PTV V_15(Gy)_ was 21.1%, below which the incidence of aRP decreased significantly. Immunotherapy has been identified as an independent risk factor for aRP.

**Conclusion:**

The occurrence of aRP in patients with lung cancer after radiotherapy has a clear dose dependency, with lungs‐PTV, V_15(Gy)_ being the best dose parameter for prediction, and the optimal cutoff value based on this study was 21.1%.

## Graphical abstract

V_15(Gy)_≤21% significantly reduces asymptomatic radiation pneumonitis (aRP) risk in lung cancer patients post‐radiotherapy. Immunotherapy independently increases aRP incidence, highlighting the need for optimized dose constraints and personalized treatment strategies.

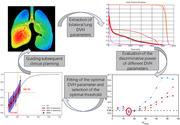

## 1 INTRODUCTION

Radiation pneumonitis (RP) is a common complication of thoracic radiotherapy, characterized by pulmonary inflammation and tissue damage. While symptomatic RP is well documented, asymptomatic radiation pneumonitis (aRP) often goes unnoticed despite its potential impact on lung function and imaging findings. A few patients may progress to RP, characterized by alveolar cell shedding and the accumulation of protein‐rich fluid in the air spaces, leading to symptoms such as cough, dyspnea, fever, and chest pain.^1^, ^2^ Severe RP is usually fatal, and according to previous research, the mortality rate of patients with non‐small cell lung cancer with severe symptoms is close to 50%.^3^

To reduce the incidence of RP and enhance treatment efficacy and patient prognosis, it is imperative to elucidate the correlation between dosimetric parameters in radiotherapy and the onset of RP. In clinical radiotherapy practice, the dose‐volume histogram (DVH) is widely used for evaluating radiotherapy plans, and V_20(Gy)_ (the percentage of lung volume receiving a dose of ≥20 Gy) is commonly used as a parameter for evaluating treatment plans. A retrospective analysis by Graham MV^4^ confirmed that V_20(Gy)_ is a risk factor for RP. The results showed that when V_20(Gy)_ was <22% and >40%, the incidence rates of RP (grade 2) were 0.7% and 36%, respectively. A study suggested that maintaining V_20(Gy)_ < 30% and a mean lung dose <20 Gy effectively limits the incidence of symptomatic radiation pneumonitis to <20%.^5^ A study by Farr KP et al.^6^ confirmed that the incidence of RP increased with an increase in V_20(Gy)_. A multivariate analysis of RP during concurrent chemoradiotherapy by Tsujino K et al. showed that V_20(Gy)_ ≥26% is a risk factor for RP ≥ grade 3.^7^ Similar results can be observed in the relevant literatures.^8^, ^9^

Although dosimetric parameters, such as V_20(Gy)_ have been extensively studied for symptomatic RP, their role in predicting aRP remains underexplored. In clinical practice, early detection and intervention in RP are crucial for effective management. Therefore, examining the relationship between various dosimetric parameters and the incidence of aRP is imperative to develop more precise and personalized radiotherapy strategies. In this study, we performed a retrospective analysis to evaluate the incidence of aRP after radiotherapy in a cohort of 72 patients with lung cancer. A statistical analysis revealed that the V_15(Gy)_ parameter was a significant predictor of aRP risk. These findings provide important insights for enhancing patient outcomes, optimizing radiotherapy protocols, and improving the precision and safety of radiation treatments.

## 2 MATERIALS AND METHODS

### 2.1 Study Cohort and Inclusion Criteria

**FIGURE 1:**
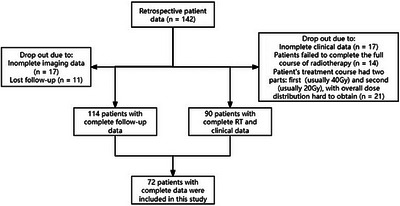
Data collection process flow chart

Among 142 patients with lung cancer receiving radiotherapy (45–65 Gy) between January 2019 and June 2022, two parallel exclusions were applied: 28 patients were excluded for incomplete follow‐up (17 lacking imaging; 11 lost to follow‐up), yielding 114 eligible patients; and 52 patients were excluded for incomplete treatment/clinical records (17 lacking clinical data, 14 undergoing incomplete radiotherapy, and 21 with split courses), yielding 90 eligible patients. The final cohort comprised 72 patients who met both criteria. The flowchart is shown in Figure 1. Ethical approval was obtained (Approval No: B2022‐518).

### 2.2 Clinical and Dosimetric Factor Generation

The following data were extracted from the patients’ medical records: demographic details (including sex, age, and smoking history), medical history (chronic obstructive pulmonary disease, diabetes mellitus, and hypertension), type of pathology, and factors related to treatment (such as chemotherapy and immunotherapy).

Computed tomography (CT) of the lungs was performed before radiotherapy. The lungs were segmented, excluding the large vessels, trachea, and proximal bronchial trees. Two additional structures were defined: lungs‐gross tumor volume (GTV), which excludes the GTV, and lungs‐planning target volume (PTV), which excludes PTV. The corresponding DVHs were produced based on the definitions of these three sets of lung structures.

The following dosimetric factors were generated based on the DVH: relative volume of lungs receiving radiation above a specified dose, denoted as V_x(%)_, with doses ranging from 5 to 55 Gy in 5 Gy increments; absolute dose to the lungs receiving more than a specified relative volume, referred to as D_x(%)_, with relative volumes ranging from 5–95% in 5% increments; absolute dose to the lungs receiving more than a specified absolute volume, indicated as D_x(cc)_, with absolute volumes ranging from 100 to 2000 cc in 100 cc increments; and the mean lung dose, represented as D_mean_.

### 2.3 Definition of aRP

The evaluation of RP was guided by the CTCAE v5.0 and the “Chinese expert consensus on diagnosis and treatment of radiation pneumonitis”.^10^ Grade 1 RP is defined as asymptomatic with clinically detectable conditions that require no intervention and is characterized by imaging changes involving <25% of the lung parenchyma. Grade 2 RP is clinically symptomatic but mild, necessitating treatment, and is characterized by extensive ground‐glass opacities beyond the irradiated area, with or without minor signs of focal consolidation involving 25%–50% of the lung parenchyma. Grade 3 RP is clinically severe, requires oxygen therapy, and poses significant evidence of focal consolidation, with or without pulmonary fibrosis, involving more than 50% of the lung parenchyma. In Grade 4 RP, life‐threatening respiratory impairment occurs, necessitating urgent intervention. In our study, the evaluation was conducted by a physician with extensive experience in chest radiotherapy. Cases with thoracic CT findings and minimal clinical intervention within the first 3 months following the completion of radiotherapy were classified as having aRP.

### 2.4 Data Processing and Statistical Analysis

To ensure comparability across different fractionation regimens, the doses were converted to an equivalent doses in 2 Gy fractions (EQD2) using a linear‐quadratic mode.^11^ This study transformed the physical dose to EQD2 using a voxel‐by‐voxel approach using the formula below^12^:

(1) $$EQD_{i}2=D_{i}\frac{\frac{D_{i}}{n}+\frac{\alpha }{\beta }}{2\mathrm{Gy}+\frac{\alpha }{\beta }}$$

$EQD_{i}2$ is the dose corresponding to voxel i after EQD2 conversion; $D_{i}$ is the physical dose of voxel i, n is the treatment fractions, and the $\frac{\alpha }{\beta }$ is 3.

We used a logistic model to fit the dose‐response relationship^13^:

(2) $$P\left(X|X_{50},\gamma_{50}\right)=\frac{1}{1+\exp\left[-4\gamma_{50}\left(\frac{X}{X_{50}}-1\right)\right]}$$

$P(X|X_{50},\gamma_{50})$ is the probability of aRP, and X is the variable of the finally selected dosimetric factors. The calculated model parameters include $X_{50}$, at which 50% of patients show a response, and the slope parameter $\;\gamma_{50}$, which is proportional to the slope of the curve at the $X_{50}$.

The Pearson chi‐square (*χ* ^2^) or Fisher's exact tests were used for a qualitative data analysis. The Mann–Whitney U test was used to analyze dosimetric data and identify the most significant factors. The forward likelihood ratio method was used to construct a multivariate logistic regression model for selecting the best indicators. Subsequently, the receiver operating characteristic (ROC) curve of the logistic regression model corresponding to the dosimetric factors was generated, and the cutoff value was determined as the maximum value of the Youden index derived from the ROC curves. Data were processed using SPSS (version 22.0; IBM, Armonk, New York, USA). All statistical tests were two‐sided; a *P*‐value ≤ 0.05 was considered statistically significant, while a *P*‐value ≤ 0.1 indicated a marginal association. EQD2 transformation and sigmoidal dose‐response fitting were performed using the programming language Python v3.11.

## 3 RESULTS

### 3.1 Characteristics of the Patient Cohort

Patients were categorized into the aRP group (30 patients) and the non‐RP group (42 patients), as illustrated in Table 1. Immunotherapy was significantly associated with an increased risk of aRP (*P* = 0.05), whereas age (*P* = 0.09) and type of pathology (*P* = 0.053) exhibited marginal associations.

**TABLE 1:** Demographic and clinical characteristics of the patient cohort and the results of the chi‐square test

Characteristics	non‐RP (*n* = 42)	aRP (*n* = 30)	*χ* ^2^	*P*‐value
Sex, *n* (%)				
Female	7 (16.667)	2 (6.667)	0.816	0.366
Male	35 (83.333)	28 (93.333)		
Age > 65 years, *n* (%)				
No	14 (33.333)	16 (53.333)	2.880	0.090
Yes	28 (66.667)	14 (46.667)		
Type, *n* (%)				
NSCLC	35 (83.333)	19 (63.333)	3.733	0.053
SCLC	7 (16.667)	11 (36.667)		
Smoke, *n* (%)				
No	19 (45.238)	13 (43.333)	0.026	0.873
Yes	23 (54.762)	17 (56.667)		
COPD, *n* (%)				
No	16 (38.095)	12 (40.000)	0.027	0.870
Yes	26 (61.905)	18 (60.000)		
Diabetes, *n* (%)				
No	38 (90.476)	27 (90.000)	‐	1.000
Yes	4 (9.524)	3 (10.000)		
Hypertension, *n* (%)				
No	20 (47.619)	17 (56.667)	0.573	0.449
Yes	22 (52.381)	13 (43.333)		
SBRT, *n* (%)				
No	35 (83.333)	28 (93.333)	0.816	0.366
Yes	7 (16.667)	2 (6.667)		
Immunotherapy, *n* (%)				
No	34 (80.952)	18 (60.000)	3.829	0.050
Yes	8 (19.048)	12 (40.000)		
Chemotherapy, *n* (%)				
No	10 (23.810)	8 (26.667)	0.076	0.783
Yes	32 (76.190)	22 (73.333)		

Abbreviations: aRP, asymptomatic radiation pneumonitis; COPD, chronic obstructive pulmonary disease; NSCLC, non‐small cell lung cancer; SBRT, stereotactic body radiation therapy; SCLC, small cell lung cancer.

### 3.2 Dose Parameter Analysis

**FIGURE 2:**
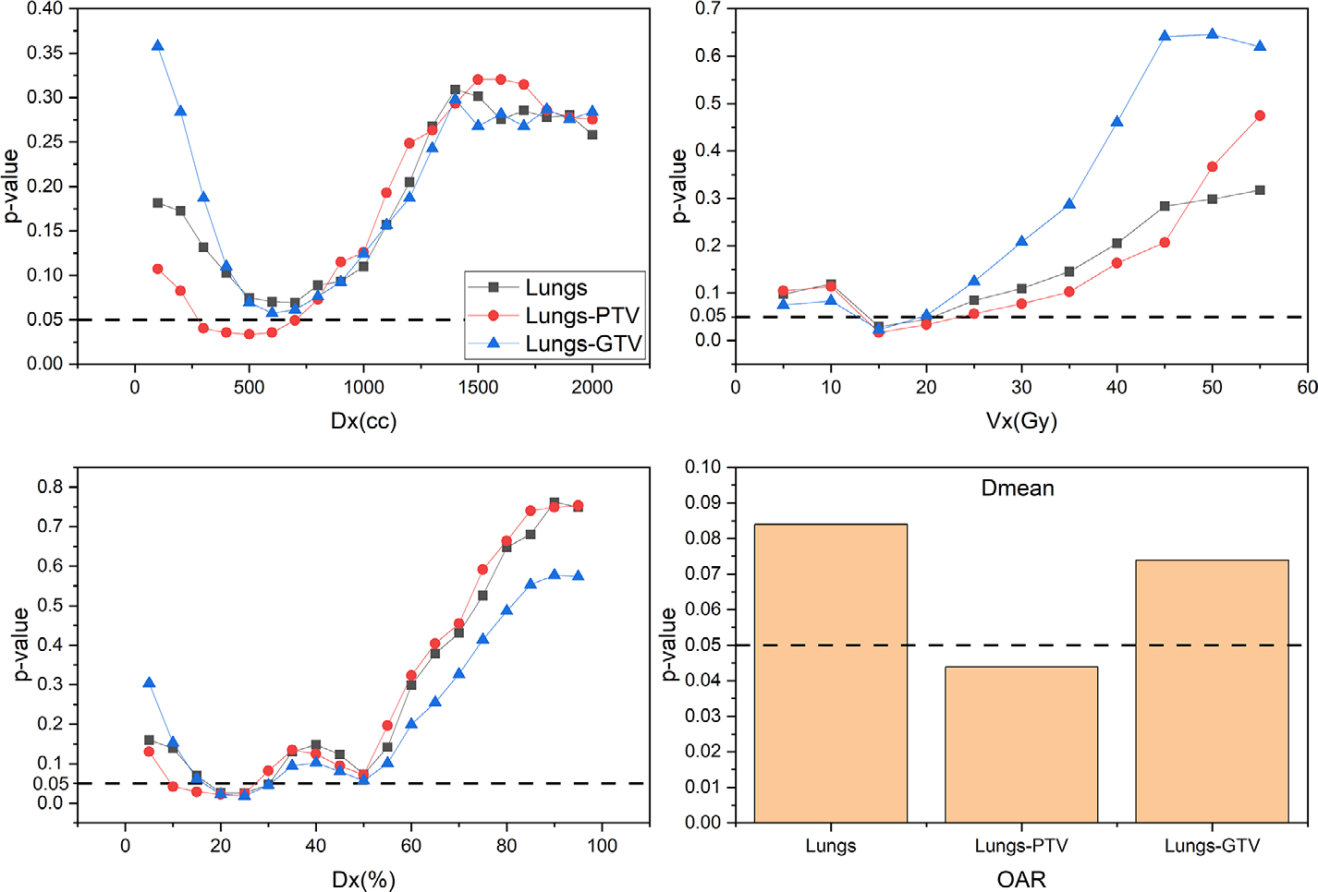
P‐values for the association between dosimetric parameters (D_x(cc)_, V_x(Gy)_, D_x(%)_, and D_mean_) and aRP risk across different lung structures (lungs, lungs‐PTV, and lungs‐GTV). Abbreviations: V_x(%)_: Relative volume of lungs receiving radiation above a specified dose; D_x(%)_: Absolute dose to the lungs receiving more than a specified relative volume; D_x(cc)_: Absolute dose to the lungs receiving more than a specified absolute volume; D_mean_: Mean lung dose

As illustrated in Figure 2, the most discriminative structure was lungs‐PTV, where D_x(cc)_ exhibited a unimodal distribution with respect to the risk of aRP occurrence. The *P*‐values were < 0.05 between D_300(cc)_ and D_700(cc)_, with D_500(cc)_ yielding the lowest *P*‐value of 0.034. Similarly, V_x(Gy)_ demonstrated a unimodal distribution, with *P*‐values < 0.05 between V_15(Gy)_ and V_20(Gy)_, with V_15(Gy)_ having the lowest *P*‐value of 0.017. In contrast, D_x(%)_ displayed a bimodal distribution, with *P*‐values < 0.05 between D_10(%)_ and D_25(%)_, with D_20(%)_ having the lowest *P*‐value of 0.022. In addition, for D_45(%)_ and D_50(%)_, the *P*‐values were all < 0.1, with D_50(%)_ showing the lowest value of 0.072. The *P*‐value for D_mean_ was 0.044. Finally, the dosimetric factors selected for aRP prediction were D_500(cc)_, V_15(Gy)_, D_20(%)_, D_50(%)_, and D_mean_. Given the strong correlation between these dosimetric factors, multiple logistic regression was performed, which ultimately identified V_15(Gy)_ as the most significant predictor.

### 3.3 Clinical‐Dosimetric Factors Relationship

The V_15(Gy)_ was 17.1% in patients receiving immunotherapy and 15.1% in those not receiving immunotherapy (*P* = 0.227). The V_15(Gy)_ was 17.6% for patients aged <65 years and 14.3% for those aged ≥65 years (*P* = 0.053). V_15(Gy)_ was 18.8% in patients with small cell lung cancer (SCLC) and 14.6% in patients with non‐small cell lung cancer (NSCLC) (*P* = 0.026). However, the PTV volume inside the lungs was significantly larger in patients with SCLC than in patients with NSCLC (*P* = 0.013). Immunotherapy has been identified as an independent predictor of aRP. The risk of aRP increased from 34.4% to 60% in patients receiving immunotherapy.

### 3.4 ROC Analysis of V_15(Gy)_ and Determination of Cutoff Values

**FIGURE 3:**
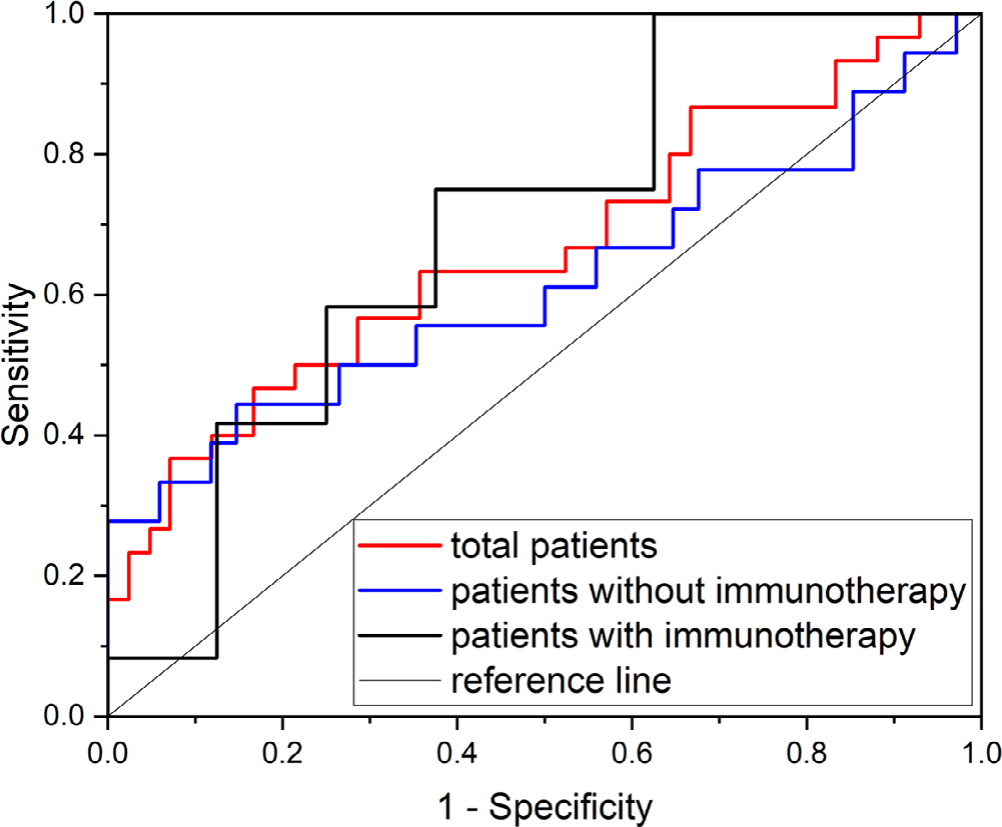
Receiver operating characteristic curves demonstrating the predictive performance of lungs‐PTV V_15(Gy)_ for asymptomatic radiation pneumonitis in the overall cohort and subgroups with or without immunotherapy

The ability of V_15(Gy)_ to discriminate aRP in the entire patient cohort, as well as in patients with or without immunotherapy, is detailed in Figure 3 and Table 2. The area under the curve (AUC) for V_15(Gy)_ was 0.666 ± 0.067 (*P* = 0.017) in the overall patient cohort. By calculating the Youden index, the optimal V_15(Gy)_ cutoff value was determined to be 21.1%. When limiting V_15(Gy)_ to ≤21%, the incidence of aRP decreased from 66.7% to 31.4%. Patients undergoing immunotherapy exhibited a lower cutoff value of 16.3%. In the absence of statistically significant differences in V_15(Gy)_, these findings indicated that immunotherapy recipients exhibit heightened radiosensitivity to aRP, thus requiring stricter V_15(Gy)_ constraints.

**TABLE 2:** Predictive performance of lungs‐PTV V_15(Gy)_ for aRP in the overall cohort and subgroups with or without Immunotherapy: AUC values and optimal cut‐off points

Type	AUC	Optimal cut‐off value
Total patients	0.666 ± 0.067 ( *P* = 0.017)	21.1%
Patients with immunotherapy	0.698 ± 0.127 (*P* = 0.143)	16.3%
Patients without immunotherapy	0.616 ± 0.090 (*P* = 0.172)	21.1%

Abbreviations: aRP, asymptomatic radiation pneumonitis; AUC, area under the curve.

### 3.5 Dose‐Response Characteristics

**FIGURE 4:**
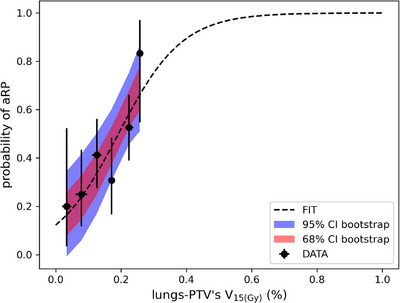
Dose–response relationship between lungs‐PTV V_15(Gy)_ and the probability of aRP in the overall patient cohort The horizontal error bars on the data points represent the standard deviation for V_15(Gy)_ within each specific bin, while the vertical error bars indicate the 68% binomial confidence interval for the observed outcomes. The dashed line represents the logistic curve. The red and blue bands denote the 68% and 95% confidence intervals, respectively, calculated using bootstrap methods. The bin sizes were set to 5% to achieve adequate resolution for visualizing the V_15(Gy)_ response while maintaining a reasonably small variance within each bin.

**FIGURE 5:**
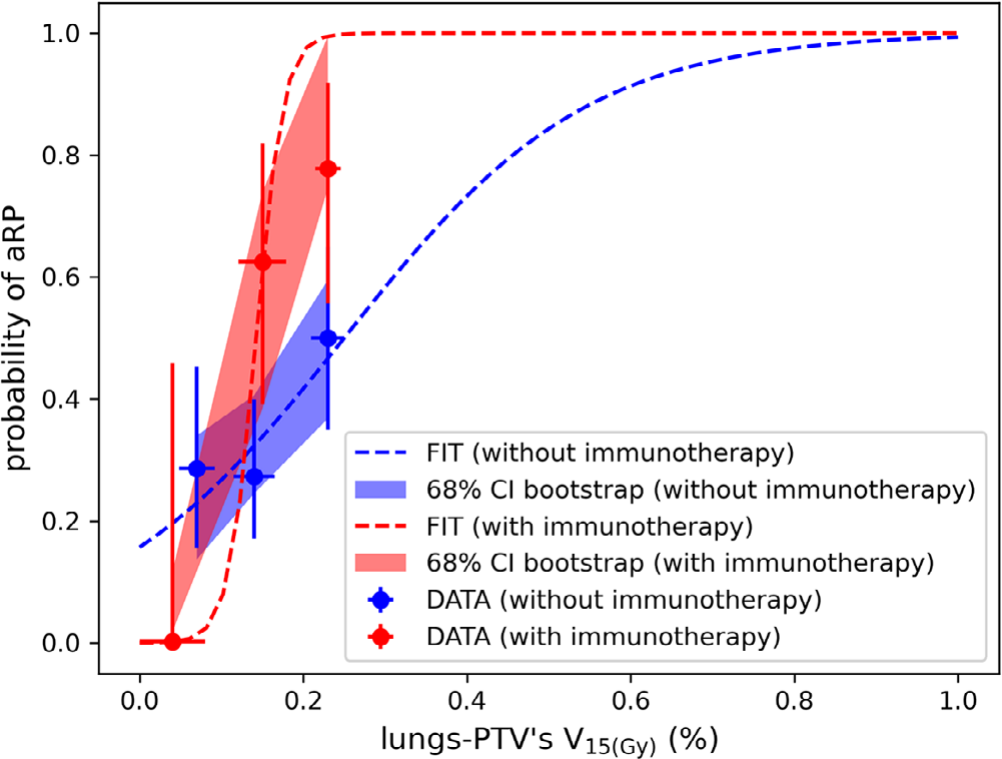
Dose–response relationship between lungs‐PTV's V_15(Gy)_ and the probability of aRP in patients with or without immunotherapy The red and blue bands, derived from bootstrap analysis, represent the 68% confidence intervals for patients with or without immunotherapy, respectively. The bin sizes were set to 10% to ensure adequate resolution for visualizing the V_15(Gy )_ response while maintaining a reasonably small variance within each bin.

Based on the comparative analysis, V_15(Gy)_ of the lung‐PTV structure was selected as the dosimetric parameter for subsequent dose–response analysis. The dose–response curves for the entire patient cohort, as well as for patients with or without immunotherapy, are provided in Figures 4 and 5. A clear dose–response relationship was observed across all the patient groups, with the logistic model demonstrating a good fit (goodness of fit = 0.70, *P* = 0.54). The $\gamma_{50}$ value of 0.49 and the $V_{(15Gy)-50}$ value of 19% further support the dose dependency of aRP. Patients receiving immunotherapy exhibited a steeper dose‐response curve ($\gamma_{50}$= 2.15) and a lower $V_{(15Gy)-50}$ value (14%), indicating a heightened sensitivity to radiation dose in this subgroup. These findings collectively provide strong evidence for the dose‐dependency of aRP, particularly in relation to V_15(Gy)_.

## 4 DISCUSSION

In this study, we evaluated 72 patients with lung cancer, of whom 30 (41.7%) developed aRP. Our analysis revealed a clear dose dependency of aRP, with the V_15(Gy)_ of lungs‐PTV emerging as the most significant predictive parameter. The aRP incidence was significantly reduced when the lungs‐PTV V_15(Gy)_ was limited to ≤21%, demonstrating a strong dose‐response relationship. This finding underscores the importance of optimizing radiotherapy plans to minimize the lungs‐PTV V_15(Gy)_, thereby reducing the risk of aRP. Furthermore, dose‐dependency was particularly pronounced in patients receiving immunotherapy, highlighting the need for tailored treatment strategies for this subgroup. The AUC of 0.666 for the lungs‐PTV V_15(Gy)_‐based aRP prediction model was statistically significant (*P* = 0.017), indicating a moderate discriminative capacity. This aligns with the recognized multifactorial pathogenesis of aRP, where the dosimetric parameters alone explain only a subset of the biological variability. Future efforts should integrate the immunotherapy status, baseline pulmonary function, and temporal dose heterogeneity to enhance the robustness of the model.

Schallenkamp JM^14^ suggested that intrathoracic radiotherapy should be planned with caution when using radiotherapy techniques that deliver doses of 10–15 Gy to large lung volumes. Metha V^15^ reported that the loss of carbon monoxide diffusion ability occurred at 13 Gy. In a separate study examining the effects of radiation on lung function, Gopal R^16^ identified a dose threshold of 13 Gy as optimal for localizing areas of lung dysfunction. Our findings align with this finding, demonstrating that high V_15(Gy)_ levels lead to alterations in chest CT imaging.

Previous studies^17^, ^18^, ^19^ reported that different lung definitions may have a significant impact on the dosimetric factors in patients with lung cancer. Therefore, the selection of lung definitions should not be ignored in clinical settings, as this has been inconsistent with the findings of previous studies. In RTOG 0617,^20^ lung volume was defined as the bilateral lung volume excluding the clinical target volume. More commonly, definitions include bilateral lung volume, excluding the planned target volume (lungs‐PTV)^4^, ^21^, ^22^ and lungs‐GTV.^5^, ^23^, ^24^ Currently, the Advisory Committee on Radiation Oncology Practice of European Society of Therapeutic Radiology and Oncology(ESTRO/ACROP) guidelines recommend the use of GTV to standardize lung volume definitions across different institutions.^25^ However, the results of this study indicate that the lungs‐PTV is more effective in predicting the occurrence of aRP, which is consistent with the conclusion of a previous study.^26^

Compared to chemoradiotherapy, immunotherapy can significantly improve the overall survival and progression‐free survival of patients. Immune maintenance after chemoradiotherapy is included in the CSCO guidelines.^27^ However, the risk of developing severe pneumonia was higher in patients who received immunotherapy than in those undergoing radiotherapy alone.^28^, ^29^, ^30^ Our findings indicate that immunotherapy may increase the incidence of aRP.

This study has certain limitations. First, the sample size of 72 individuals was relatively small, which may have hindered the ability to draw meaningful conclusions. Second, as this was a retrospective study, it was subject to an inherent bias. Further prospective studies are required to validate and refine our findings. Additional limitations include the short follow‐up period and reliance on a physician's subjective assessment of RP severity, as per established guidelines. Despite these limitations, we believe that our study will contribute to the management of patients with lung cancer during radiotherapy and enhance their quality of life.

## 5 CONCLUSION

This study demonstrates that aRP incidence is dose‐dependent, with lungs‐PTV V_15(Gy)_≤21% significantly reducing its risk. Immunotherapy has been identified as an independent risk factor for aRP. Future studies should validate these findings in larger cohorts to further refine radiotherapy planning strategies.

## AUTHOR CONTRIBUTIONS

**Weixing Ji**: Methodology; formal analysis and writing of the original draft. **Tao Jiang**: Clinical evaluation of patients and assessment and diagnosis of radiation pneumonitis. **Zhihan Chen**: Software implementation and validation. **Gang Chen**: Data collection and analysis. **Yang Zhang**: Data interpretation; critical revision; review and editing. **Shisuo Du**: Supervision; project administration; writing; review and editing. All authors reviewed the manuscript and approved the final version for publication.

## CONFLICT OF INTEREST STATEMENT

The authors declare no conflict of interests.

## ETHICAL APPROVAL

Ethical approval was obtained from the Ethics Committee of Zhongshan Hospital, Fudan University (Approval No: B2022‐518). The requirement for written informed consent was waived due to the retrospective nature of the study

## Data Availability

The data supporting this study are available from the corresponding author upon reasonable request.
